# Translation of the Headache-Attributed Restriction, Disability, Social Handicap and Impaired Participation (HARDSHIP) questionnaire into the Croatian language, and its diagnostic validation

**DOI:** 10.3325/cmj.2022.63.202

**Published:** 2022-04

**Authors:** Lukrecija Jakuš, Darija Mahović, Timothy J Steiner

**Affiliations:** 1 University of Applied Health Sciences, Zagreb, Croatia; *lukrecija.jakus@zvu.hr*; 2 Department of Neurology, University Hospital Center Zagreb, Croatia; 3 University of Zagreb, School of Medicine, Zagreb, Croatia; 4 Department of Neuromedicine and Movement Science, Norwegian University of Science and Technology, Trondheim, Norway; 5 Division of Brain Sciences, Imperial College London, London, United Kingdom

Despite increasing awareness promoted by the Global Campaign against Headache (1,2) and the Global Burden of Disease (GBD) study (3-5), knowledge and understanding of the burdens attributable to headache disorders remain incomplete in many countries.

Croatia is one such country. A literature review identified eight studies conducted in Croatia between 1999 and 2015 reporting the prevalence, incidence, and/or frequency of migraine, tension-type headache (TTH), or disorders characterized by headache occurring on ≥15 days/mo (H15+) among adolescents or adults (6). Major methodological differences between these studies, and variable quality, make comparisons difficult (6). Most adopted appropriate cross-sectional designs (7-13) but not all used (or reported) adequate methods of sampling from the population of interest. Most applied diagnoses according to modifications of the International Classification of Headache Disorders (ICHD) criteria, either ICHD-II (14) or the earlier ICHD-I (15), but none reported validation of the diagnostic enquiry. Many reported prevalence and some reported symptoms and individual burden, but none reported burden on others or societal burden. One was observational, individual, and uncontrolled, limited to medical students (16). Three cross-sectional studies examined the prevalence of headaches among adolescents (7-9). Four studies reported headache among adults (10-13), of which two were population-based using door-to-door sampling and face-to-face interviews, but were limited to adult residents of Bakar (a small town in Primorsko-Goranska County with about 8000 inhabitants) (12,13). One reported one-year prevalence of migraine, probable migraine, and TTH in an adult population sample of four Croatian cities (Zagreb, Split, Osijek, and Dubrovnik) using a self-completed questionnaire (11). One was focused on “chronic headache” (10).

## HARDSHIP QUESTIONNAIRE

Lifting The Burden (LTB), the UK-registered non-governmental organization leading the Global Campaign against headache in official relations with the World Health Organization (1,2,17), has supported population-based burden-of-headache studies in many countries (18), developing, for the purpose, standardized methodology (19,20) and a survey instrument (the Headache-Attributed Restriction, Disability, Social Handicap and Impaired Participation [HARDSHIP] questionnaire) (21). In a modular format, HARDSHIP includes demographic enquiry, headache screening questions, diagnostic enquiry based on ICHD-3 criteria (22), and questions on several quantifiable components of headache-attributed burden (symptoms, disability, lost productive time, personal financial burden, cumulative burden, health-resource consumption, and burdens on others including societal economic burden) (21). It is designed for data collection by trained lay interviewers in face-to-face enquiry, who are not expected to diagnose headache type (migraine, TTH, H15+ including probable medication-overuse headache [MOH]; this is done algorithmically from the diagnostic question set (21). The sensitivity and specificity of this question set for migraine and TTH have been demonstrated in multiple cultures, so far in 20 countries and 19 languages (21).

In a pilot study undertaken as a prerequisite for a cross-sectional study of the prevalence and burden of primary headache disorders among University of Applied Health Sciences students in Zagreb, Croatia, we culturally adapted the HARDSHIP questionnaire and translated it into the Croatian language. We then validated the headache screening and diagnostic questions in two stages: first in a patient sample in clinical settings and second in a convenience sample drawn from the population of interest.

The study was approved by the Ethics Committee of the University Hospital Center Zagreb and the Ethics Committee of the University of Applied Health Sciences. Informed consent was obtained from all participants. Data protection legislation was complied with.

## Cultural adaptation and translation of HARDSHIP

We translated HARDSHIP from the original English into Croatian in accordance with LTB’s translation protocol for hybrid documents (23). In the process, we made cultural adaptations as necessary.

Translation was coordinated by LJ, a native Croatian speaker and resident of Croatia, bilingual in English and with technical understanding of HARDSHIP questions. Forward-translations were prepared by DM, a similarly qualified headache expert, and by a professional translator without technical knowledge. Discrepancies in the forward translations were resolved between the coordinator and translators until consensus was reached. One back-translation of the final forward translation was carried out by an occupational therapy professional, a native speaker of English, bilingual in Croatian, blinded to the purpose of the questionnaire. The back-translation and report of the translation procedure were reviewed by TJS, who compared the original and back-translated versions for conceptual equivalence. The few observed discrepancies were resolved by the forward translators and coordinator. Finally, a target audience review (face validation) was carried out by interviewing six conveniently available persons aged 20-43: four students (two male and two female) and two lecturers (physical therapist and psychologist) from the University of Applied Health Sciences. All were positive to the screening questions: “Have you ever in your life had a headache?” and “Have you had a headache in the last 12 months?” They were asked whether the wording of questions was comprehensible and culturally appropriate.

Adaptations were needed only with respect to total annual net household income quintiles and to medications available for treatment of headache. We do not report the latter. For the former we established the average disposable income per household in 2018, in Croatian Kuna, as HRK 97 870 (USD 1 ~ HRK 6.4). As an indicator of poverty and social exclusion, the at-risk-of-poverty threshold for a household consisting of two adults and two children was HRK 62 622 (24). As an indicator of inequality of income distribution, the 20% of the population with the highest equivalized disposable income earned 5 times the 20% with the lowest equivalized disposable income. Table 1 shows the total annual net household income quintiles estimated accordingly, which will be applied to categorize participants’ socioeconomic status in the cross-sectional study of headache prevalence and headache-attributed burden.

**Table 1:** Total annual net household income quintiles*

Quintile	Category	Income range (HRK)
1	very poor	<30 000
2	poor	30-70 000
3	intermediate	70-110 000
4	wealthy	110-150 000
5	very wealthy	>150 000

*Data from Croatian Bureau of Statistics Indicators of Poverty and Social Exclusion, 2018 (24).

The target audience review found the wording of all translated questions comprehensible and appropriate. The sample of 32 headache patients found the questions culturally inoffensive and the length of the questionnaire acceptable. Feedback from the diagnostic validation prompted no changes. Thus, the translation procedure was completed according to protocol (23) and a Croatian version of HARDSHIP was developed.

## Diagnostic validation of Croatian version of HARDSHIP questionnaire

First, in a preliminary validation exercise, we took a small convenience sample of 32 headache patients from the headache center at Department of Neurology, University Hospital Center Zagreb. Questionnaire-derived diagnoses were observationally compared with diagnoses made at clinical interview and examination by the headache expert.

Second, we performed diagnostic validation in a subsample of 150 participants in the survey of University of Applied Health Sciences students. In this survey, to be reported later, the questionnaire was administered to participants in small groups of 15 to 20 in the university setting. After the investigator had described the questionnaire in detail to each group, and provided opportunity for clarifications, each student self-completed the questionnaire. The subsample, all native Croatian speakers, were consecutively selected according to their questionnaire-derived diagnoses: 30 reporting no headache, 75 with migraine (either definite or probable), and 35 with TTH (either definite or probable), plus 10 with headache unclassifiable by questionnaire. All were invited, within 10 days of completing the questionnaire, to re-interview and examination by a headache expert blind to the questionnaire diagnoses.

Definite and probable migraine were grouped together, as were definite and probable TTH (21). We calculated sensitivity, specificity, and positive (PPV) and negative (NPV) predictive values (25) for migraine and TTH taking the headache expert’s diagnoses as reference. We calculated Cohen’s kappa (κ) with 95% confidence interval for the overall agreement between questionnaire-derived diagnoses and headache expert’s diagnoses.

Of the 150 participants selected for the validation subsample, five refused: one reporting no headache, two diagnosed with migraine, and two unclassifiable by questionnaire. Of the 145 participants re-interviewed by a headache expert (130 female; mean age 22.9 ± 2.0 years), 29 reported no headache, 73 were diagnosed with migraine by the questionnaire, 35 with TTH, and eight were unclassifiable by questionnaire. The expert diagnosed no headache in 29 cases (full match), migraine in 68, TTH in 37 cases, and other headache in 11 (10 secondary headaches and one cluster headache).

No major problems were experienced. A small convenience sample drawn from patients in clinic found the questions culturally inoffensive, and the length of the questionnaire acceptable. For the screening questions, we found 100% agreement in headache caseness vs non-caseness (no headache in the preceding year) in the validation subsample of N = 145. For the diagnostic questions, we found very high sensitivity (95%) and high specificity (89%) for migraine, with PPV of 89%, NPV of 95%, and κ = 0.85 (very good agreement). For TTH we found somewhat lower sensitivity (78%), very high specificity (94%), PPV of 83%, NPV of 93%, and κ = 0.74 (good agreement). The Croatian-language version of HARDSHIP is validated and ready to use in epidemiological studies.

There is one caveat: the validation was not performed in a general-population sample but in a subsample of university students, who were the subject of further enquiry. This suited our purpose, but it was a study limitation in that the participants were highly educated and able to give good accounts of themselves. Our findings may not be generalizable to a broader spectrum of respondents.

The HARDSHIP questionnaire has been validated in many languages (26-29), in each case against reference diagnoses made by headache experts. While questionnaire-derived diagnoses separated definite and probable migraine, and definite and probable TTH, we followed previous authors in combining definite and probable cases of each for purposes of validation (26-29). “Probable” diagnoses, as defined by ICHD (22), serve a purpose in clinical management pending confirmation during follow-up, but not in epidemiological surveys.

The previous validation studies, in Mandarin (26), Russian (27), Kannada (28) and Urdu (29), demonstrated agreement, from fair/substantial to very good, between questionnaire and experts’ diagnoses for migraine, and from slight to fair/substantial agreement for TTH. Judged by κ values, our Croatian translation performed as well as or better than any, but this may reflect the nature of our respondents. There are several reasons why agreement is never perfect, and unreliable recounting by respondents on different occasions, even when less than 10 days apart, can be one. A factor to be considered here is that HARDSHIP instructs respondents to focus on the most bothersome headache when they report headaches of more than one type. This is to avoid mixed accounts, but it is not known how well they do this. The subjective perception of what is most bothersome may vary, perhaps influenced by whichever headache occurred most recently. Without biological markers, expert diagnoses are not the “gold standard” they are often proclaimed to be; they are opinions (albeit expert ones) formed during brief single interviews. In the course of clinical management, these would be reviewed. Nevertheless, structured questionnaires are inherently limited in their scope of enquiry, whereas experts expand and seek clarification. Finally, κ values have a number of influencers (including prevalence); as statistical measures of chance-corrected agreement, they should be regarded, and interpreted, with a certain circumspection (30).

Our study had a few other limitations. The refusal of re-interview by five (3.4%) of the selected respondents was unlikely to be influential, but participants were mostly female (90%), with a high proportion of migraine cases. The validation study focused on primary headache disorders and did not include secondary headaches (none of the sample were diagnosed with MOH).

In conclusion, the Croatian-language version of HARDSHIP diagnostic questionnaire was validated in university students and performed well. In other languages, and even in countries with high levels of illiteracy, HARDSHIP has proved reliable for the purpose of epidemiological surveys (31-39).
